# The Moderating Effect of Generation on the Association Between Long Working Hours and Mental Health: A Cross-Sectional Study of Korean Employees

**DOI:** 10.3390/healthcare13233002

**Published:** 2025-11-21

**Authors:** Sra Jung, Yoosuk An, Sang-Won Jeon, Junhyung Kim, Eunsoo Kim, Jeong Hun Yang, Sung Joon Cho

**Affiliations:** 1 Department of Psychiatry, CHA University Ilsan CHA Hospital, Goyang-si 10313, Republic of Korea; srajung890@chamc.co.kr; 2 Department of Psychiatry, National Traffic Injury Rehabilitation Hospital, Yangpyeong 01022, Republic of Korea; ensonic@gmail.com; 3 Department of Neuropsychiatry, Seoul National University Hospital, Seoul 03080, Republic of Korea; 4 Department of Psychiatry, Seoul National University College of Medicine, Seoul 03080, Republic of Korea; 5 Department of Psychiatry, Kangbuk Samsung Hospital, Sungkyunkwan University School of Medicine, Seoul 03181, Republic of Korea; sangwonyda@hanmail.net (S.-W.J.); jihndy.kim@samsung.com (J.K.); silverstream421@gmail.com (E.K.); 6 Workplace Mental Health Institute, Kangbuk Samsung Hospital, Seoul 03181, Republic of Korea; 7 Department of Psychiatry, Chungnam National University Sejong Hospital, Sejong-si 30099, Republic of Korea; 8 Department of Statistics and Data Science, Korea National Open University, Seoul 03087, Republic of Korea

**Keywords:** workplace mental health, long working hours, depression, anxiety, generational differences

## Abstract

**Background/Objectives:** Long working hours are a recognized risk factor for poor mental health, but their impact may vary across generations. Millennials and Generation Z (MZ generation) have received attention for their distinct values, work–life expectations, and communication styles compared with older cohorts. The present study assessed generational differences (MZ vs. non-MZ) in the association between weekly working hours and depressive and anxiety symptoms among Korean employees. **Methods:** We analyzed cross-sectional data from 11,372 employees (68.0% men; mean age = 36.7 years) who underwent mental health screenings between 2020 and 2022. Participants born on or after 1 January 1980, were classified as belonging to the MZ generation (*n* = 7538). Symptoms of depression and anxiety were assessed using the Center for Epidemiologic Studies Depression Scale (CES-D) and the Clinically Useful Anxiety Outcome Scale (CUXOS). Multiple linear regression models tested the interaction between mean-centered weekly working hours and generation, adjusting for sociodemographic covariates. **Results:** Longer working hours were significantly associated with greater depressive (B = 0.080, *p* < 0.001) and anxiety (B = 0.119, *p* < 0.001) symptom severity. Significant interactions between generation and working hours were observed for both depression (B = 0.140, *p* < 0.001) and anxiety (B = 0.182, *p* < 0.001). Generation-stratified models showed that the increase in symptom severity per additional work hour was approximately three-fold higher in the MZ generation than in the non-MZ generation. **Conclusions:** The mental health burden of long working hours is amplified in the MZ generation. Generation-sensitive workplace health strategies are needed to address this vulnerability.

## 1. Introduction

The work environment is a key determinant of daily life and mental health [1]. When job-related stressors exceed an individual’s adaptive capacity, they can lead to various mental health problems, including symptoms of depression and anxiety [2,3]. These symptoms contribute to absenteeism, reduced work efficiency, and presenteeism—a state of being physically present at work but unable to perform effectively. Such consequences not only harm individual well-being but also lower organizational productivity and impose a substantial economic burden on society [4]. Therefore, protecting workers’ mental health and creating healthy work environments require identifying key stress factors and understanding their underlying mechanisms [5].

Among workplace stressors, long working hours have consistently been identified as one of the most significant threats to employees’ mental health [6,7]. Excessive working hours deprive individuals of opportunities for rest and recovery, disrupt home and social life, and undermine work–life balance [8]. This state of chronic strain and resource depletion increases the risk of negative mental health outcomes, including burnout, depression, and anxiety [9,10]. Despite ongoing legal efforts to shorten work hours, South Korea continues to record some of the longest working hours worldwide. According to the OECD Employment Outlook (2024) [11], Korean employees worked an average of 1865 h per year in 2023—well above the OECD average of 1736 h—highlighting the persistence of this public health issue making this an enduring public health concern [12,13,14].

However, not all generations respond to long work hours in the same way. In today’s organizations, multiple generations coexist, and studies have shown generational differences in work-related values, expectations, and stress coping strategies [15]. Millennials and Generation Z (MZ), who now form the backbone of the workforce, hold distinct values compared with older cohorts [16]. For example, they place greater emphasis on personal growth, meaningful work, fair compensation, and work–life balance, while showing less adherence to traditional values such as organizational loyalty and job security [17]. They also prioritize horizontal communication and flexibility over rigid hierarchical cultures, and they exhibit greater psychological resistance to excessive work demands that infringe on their personal lives [18]. These generational characteristics suggest that MZ workers may be more vulnerable to long work hours, which may not only extend physical time demands but also directly challenge their core values [19]. Supporting this idea, a study comparing generations in the nursing profession reported that burnout was three-fold more prevalent among Millennials compared with Generation X, and six-fold more prevalent compared with Baby Boomers [19].

Previous research has indicated that the effects of workplace stress on mental health can be moderated by various personal characteristics, such as sex and occupational rank [9,12,20]. From this perspective, “generation,” associated with distinct work-related values and stress perceptions, may represent another important moderator of the relationship between long working hours and mental health. Some qualitative studies have reported that long working hours are closely related to occupational burnout and depression among MZ workers [21,22]. However, few large-scale empirical studies have statistically validated this potential moderating effect [23].

Accordingly, this study aimed to use a large sample of South Korean employees to investigate whether generation (MZ vs. non-MZ) moderates the association between work hours and symptoms of depression and anxiety. By providing robust empirical evidence, we sought to inform customized workplace strategies and policies tailored to generational characteristics. The specific hypotheses of this study were as follows:

**Hypothesis** **1.** *Longer work hours will be significantly positively associated with depressive and anxiety symptoms.*

**Hypothesis** **2.** *The positive association between long work hours and depressive symptoms will be significantly stronger among MZ workers compared with non-MZ workers.*

**Hypothesis** **3.** *The moderating effect of generation will be specific to depressive symptoms and not significant for anxiety symptoms.*

By clarifying generational differences in vulnerability to long working hours, this study aims to contribute to the development of generation-specific strategies to improve workplace mental health.

## 2. Materials and Methods

### 2.1. Participants

Data were obtained from 11,372 working adults aged 19–65 years who completed standardized mental health evaluations at the Workplace Mental Health Institute, Kangbuk Samsung Hospital (Seoul, South Korea) during April 2020–March 2022. These participants were employed by one of 18 large corporations or public institutions and voluntarily took part in mental health screening programs provided by their organizations.

From an initial pool of 12,361 individuals, we excluded those with missing data on key study variables, including weekly working hours, depressive and anxiety symptom scores, demographic characteristics, or generation classification. The final analytic sample consisted of 7737 men and 3635 women.

Approval for this research was granted by the Institutional Review Board of Kangbuk Samsung Hospital (KBSMC 2022-03-046). The study complied with the ethical principles of the Declaration of Helsinki and Good Clinical Practice. Since only de-identified health screening records were analyzed, written informed consent was waived.

### 2.2. Clinical Assessments

Participants completed structured self-administered questionnaires that included items on age, sex, educational attainment (college graduate or below, university graduate, and master’s degree or higher), marital status (married, unmarried, other), years of service in the current workplace, average weekly working hours, and monthly income (<3 million Korean Won [KRW], 3–4 million KRW, >4 million KRW). According to Statistics Korea (2023) [24], the mean monthly salary of Korean employees was approximately 3.63 million KRW; thus, the 3–4 million KRW range was considered the average income category, with values below and above representing low- and high-income groups, respectively. Missing income data were coded as a separate category to retain the full sample.

Generation group was defined by date of birth: individuals born before 1 January 1980, were classified as non-MZ, and those born on or after this date were classified as MZ generation [25].

Depressive symptom severity was measured using the 20-item Korean version of the Center for Epidemiologic Studies Depression Scale (CES-D) [26,27], which assesses a 20-item self-report questionnaire assessing affective, somatic, and interpersonal aspects of depression during the past week. Items are rated on a 4-point Likert scale (0–3), yielding total scores of 0–60, where higher scores indicate greater symptom severity. Internal consistency (Cronbach’s alpha) for the CES-D in the current sample was 0.765.

Anxiety symptoms were assessed using the Korean version of the Clinically Useful Anxiety Outcome Scale (CUXOS) [28,29], a 20-item self-administered questionnaire measuring the severity of both psychic and somatic anxiety symptoms over the past week, including the day of assessment. Items were scored on a five-point scale from 0 (“not at all true”) to 4 (“almost always true”), giving a total range of 0–80. Higher scores correspond to increased anxiety severity. The CUXOS showed high internal reliability in this sample (Cronbach’s α = 0.952).

Both instruments have validated Korean versions and are widely used in occupational and community mental-health studies. They were chosen because they provide continuous severity measures rather than categorical screening, allowing for sensitive detection of dose–response relationships between weekly working hours and mental-health outcomes. Their broad coverage of affective, somatic, and interpersonal domains also enhances comparability with prior large-scale epidemiologic research.

### 2.3. Statistical Analysis

Descriptive statistics were calculated to compare sample characteristics between the MZ and non-MZ generations. Independent-sample *t*-tests were applied to continuous variables, and chi-square tests were used for categorical variables.

To examine whether the association between weekly working hours and mental health outcomes differed by generation, we performed multiple linear regression analyses with CES-D and CUXOS total scores as dependent variables in separate models. Model 1 included centered weekly working hours and sociodemographic and occupational covariates (age, education level, marital status, years of service, and income category). Model 2 further added the generation group and the interaction term between generation and centered weekly working hours to test moderation effects within a single integrated model rather than by comparing coefficients across stratified models. Because generation and chronological age capture related but distinct constructs (cohort-related psychosocial characteristics vs. biological aging) and are inherently collinear, we specified generation (MZ vs. non-MZ) as the primary moderator in the main models. As a robustness check, we re-estimated the models substituting continuous age for generation; the Age × Working Hours interaction remained significant with a comparable increase in explained variance (ΔR^2^ ≈ 0.015–0.018), indicating that the moderation effect is not dependent on a particular operationalization. To further explore whether the moderating effect of generation differed by sex or marital status, exploratory three-way interaction terms (Generation × Working Hours × Sex and Generation × Working Hours × Marital Status) were tested in separate regression models, adjusting for the same covariates as in the main analyses. These analyses aimed to examine potential gender- and relationship-related variations in the generation-specific impact of long working hours on mental health. To further explore subgroup variations in the moderating effect of generation, we conducted supplementary analyses stratified by sex, marital status, and income category. Predicted CES-D and CUXOS scores for each one-hour interval of weekly working hours were computed based on the extended regression models, and the results were visualized.

Because this was a cross-sectional, observational dataset without a defined treatment or exposure group, propensity-score weighting was not applied; instead, potential confounding was addressed by including relevant covariates directly in the regression models. Regression diagnostics, including variance-inflation-factor (VIF) analysis, confirmed the absence of problematic multicollinearity. Data analyses were performed using IBM SPSS Statistics, version 28.0 (IBM Corp., Armonk, NY, USA). Statistical significance was defined as a two-sided *p* < 0.05.

## 3. Results

### 3.1. Baseline Demographic Characteristics

Of the 11,372 participants included in the analysis, 3834 (33.7%) were classified as non-MZ and 7538 (66.3%) as MZ based on birth year. The overall mean age was 36.72 ± 9.43 years; non-MZ participants were substantially older than MZ participants (47.70 ± 5.08 vs. 31.14 ± 5.34 years, *p* < 0.001). Sex distribution also differed significantly between groups (*p* < 0.001): men comprised a larger proportion of the non-MZ group (72.4% vs. 65.8%), whereas women were more represented among MZ participants (34.2% vs. 27.6%). Educational attainment varied by generation (*p* < 0.001). A greater proportion of MZ participants held a university degree (64.8% vs. 50.9%), whereas non-MZ participants were more likely to have attained a master’s degree or higher (25.5% vs. 10.6%). Marital status also differed markedly (*p* < 0.001). Nearly nine in ten non-MZ participants were married (88.4%) compared with only 39.5% of MZ participants. Conversely, 59.6% of MZ participants were unmarried compared with 8.2% in the non-MZ group.

Regarding occupational characteristics, non-MZ participants had markedly longer tenure at their current workplace (19.10 ± 9.57 vs. 6.20 ± 4.68 years, *p* < 0.001). Average weekly working hours were slightly but significantly lower in the non-MZ group (46.14 ± 8.34 vs. 47.17 ± 7.06 h, *p* < 0.001). Monthly income distribution also differed significantly (*p* < 0.001): 67.1% of non-MZ participants earned more than 4 million KRW per month compared with 27.7% of MZ participants, whereas 34.4% of MZ participants earned less than 3 million KRW compared with 9.2% of non-MZ participants.

Regarding depressive symptoms, the MZ generation exhibited significantly higher mean CES-D scores compared with the non-MZ generation (14.99 ± 10.16 vs. 13.50 ± 9.63, *p* < 0.001). CUXOS scores did not differ significantly between generations (17.19 ± 14.82 vs. 16.85 ± 14.14, *p* = 0.230) (Table 1).

### 3.2. Association Between Weekly Working Hours, Generation, and Depressive Symptoms

In the base model (Model 1), being a woman (B = 3.834, *p* < 0.001), having a university education compared with college or below (B = 1.255, *p* < 0.001), unmarried status (B = 1.629, *p* < 0.001), and other marital status (B = 3.827, *p* < 0.001) were significantly associated with higher CES-D scores. Monthly income above 4 million KRW was associated with lower depressive symptoms compared with income below 3 million KRW (B = −0.560, *p* = 0.044).

**Figure 1:**
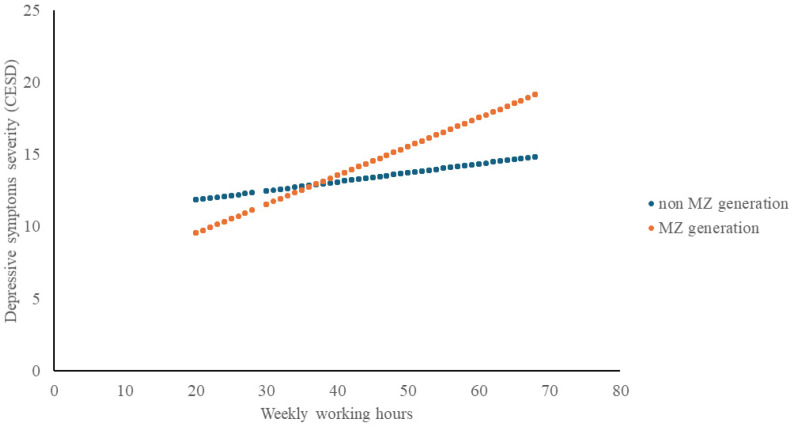
Working hours and depressive symptoms (CES-D) by generation.

In the augmented model (Model 2), which accounted for generation and its interaction with weekly working hours, greater working hours were linked to higher depressive symptom scores (B = 0.080, *p* < 0.001). Belonging to the MZ generation was also associated with higher CES-D scores (B = 1.942, *p* < 0.001). Importantly, the interaction between MZ generation and weekly working hours was significant (B = 0.140, *p* < 0.001). Model 2 explained a greater proportion of variance in depressive symptoms than Model 1 (R^2^ = 0.071 vs. 0.051) (Table 2, Figure 1).

Generation-stratified analyses reinforced this pattern (Table S1). In the non-MZ group, each additional weekly working hour was associated with a 0.064-point increase in CES-D score (*p* < 0.001), whereas in the MZ group, the corresponding increase was more than three-fold higher (B = 0.223, *p* < 0.001). Being a woman, unmarried or other marital status, and longer years of service were significant predictors in both groups, while higher income (>4 million KRW) was protective.

As a robustness check, we conducted an additional analysis replacing generation with continuous age while retaining the same covariates. The interaction between age and weekly working hours was also significant (B = −0.007, *p* < 0.001), and the increase in explained variance (ΔR^2^ = 0.017) was comparable to that of the generation-based model. This consistency supports that the moderating effect is not merely attributable to chronological age but reflects broader cohort-related differences.

### 3.3. Association Between Weekly Working Hours, Generation, and Anxiety Symptoms

In the base model (Model 1), older age (B = 0.098, *p* < 0.001), being a woman (B = 6.783, *p* < 0.001), and having a university education compared with college or below (B = 2.273, *p* < 0.001) were significantly associated with higher CUXOS scores. Other marital status was also linked to greater anxiety severity (B = 2.616, *p* = 0.011), while monthly income over 4 million KRW was associated with lower scores (B = −1.692, *p* < 0.001).

**Figure 2:**
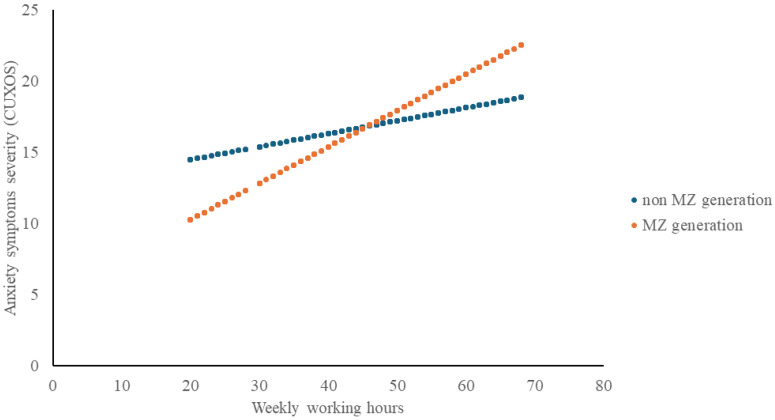
Working hours and anxiety symptoms (CUXOS) by generation.

The extended regression model (Model 2) revealed a positive relationship between weekly working hours and anxiety severity (B = 0.119, *p* < 0.001). Belonging to the MZ generation was also associated with higher anxiety severity (B = 2.526, *p* < 0.001). The interaction between generation and weekly working hours was significant (B = 0.182, *p* < 0.001). Adding these variables increased the explained variance from 6.3% to 8.0% (Table 3, Figure 2).

Generation-stratified analyses confirmed this pattern (Table S2). In the non-MZ group, each additional weekly working hour was associated with a 0.100-point increase in CUXOS score (*p* < 0.001), whereas in the MZ group, the corresponding increase was approximately three-fold larger (B = 0.305, *p* < 0.001). Being a woman remained a strong predictor in both groups, with a larger effect in the MZ group (B = 7.425 vs. B = 5.055). University education was significant only among MZ participants (B = 2.292, *p* < 0.001). Higher monthly income over 4 million KRW was protective in both groups, and marital status was associated with anxiety only among non-MZ participants.

To verify the robustness of this pattern, we re-estimated the models using continuous age instead of generation. The interaction between age and weekly working hours remained significant (B = –0.008, *p* < 0.001), and the gain in explained variance (ΔR^2^ = 0.016) was almost identical to that observed in the generation-based analysis. These results suggest that the moderating effect of generation is not simply an age artifact.

### 3.4. Exploratory Three-Way Interaction Analyses

To examine whether the moderating effect of generation differed by sex, we included a Generation × Working Hours × Sex term in the regression models. The three-way interaction approached significance for both depressive (B = 0.057, *p* = 0.081) and anxiety symptoms (B = 0.080, *p* = 0.094), with the steepest slopes observed among MZ women, suggesting a possible gender-specific vulnerability.

When marital status was examined as an additional moderator, the Generation × Working Hours × Marital Status interaction was not statistically significant (*p* = 0.502 and 0.477 for depressive and anxiety models, respectively). However, both Generation × Working Hours and Generation × Marital Status two-way interactions remained significant, indicating that MZ workers experienced greater adverse effects of long working hours and more pronounced marital-status–related differences in depressive and anxiety symptoms compared with non-MZ workers.

To further illustrate these patterns, subgroup-specific predicted scores were computed for sex, marital status, and income categories and are presented in Supplementary Figures S1–S6.

## 4. Discussion

This study is the first to use a large-scale, multicenter sample of more than 11,000 Korean workers to quantitatively examine how the relationship between long working hours and mental health differs between generations. Long working hours significantly exacerbated both depressive and anxiety symptoms, and these negative effects were markedly stronger in the MZ generation than in the non-MZ generation. In particular, there was a clear interaction between weekly working hours and mental health outcomes: the same increase in work hours was associated with a three-fold greater exacerbation of symptoms in the MZ generation. The academic and practical value of this study lies in its use of large-scale data to verify differences in vulnerability to work stress between the MZ and earlier generations.

Considering the sociodemographic characteristics in this study, the MZ generation is, by definition, younger, with fewer years of work experience and lower economic stability. The proportion of women participants was higher among the MZ generation, which may reflect both discontinuity in employment among women of previous generations because of childbirth and the effects of recent policies promoting female employment. Marriage rates and income levels were also lower among MZ participants. These differences may partly reflect age, but they also suggest broader generational influences such as housing affordability, economic conditions, and the tendency to delay family formation. Regarding education, although the proportion of junior college graduates was similar across groups, MZ participants were relatively more likely to hold university degrees, while non-MZ participants were more likely to have pursued postgraduate study. This may be linked to the so-called culture of “small but certain happiness” in South Korea, whereby younger generations prioritize immediate experiences and ‘realistic satisfaction over accumulating academic credentials for the future’ [30]. By contrast, older generations often pursued postgraduate study alongside employment to remain competitive for long-term promotions.

In this study, CES-D scores were significantly higher among MZ participants, whereas CUXOS scores did not differ by generation. Several psychosocial factors may explain this finding. MZ individuals are more likely to experience learned helplessness, as they often face limited rewards despite substantial effort in the context of structural challenges such as a stagnant economy, employment instability, and rising housing costs [31,32]. Growing up under the high expectations of Baby Boomer and Gen X parents may also have contributed to helplessness and despair through repeated experiences of falling short of achievement-based standards [33]. In addition, overprotective or intrusive parenting can hinder the development of independent problem-solving skills and increase vulnerability to depression [34]. Furthermore, as the first generation to grow up with social networking services (SNS) as an everyday phenomenon, MZ participants are frequently exposed to upward social comparisons and the perceived happiness of others, fostering feelings of relative deprivation and chronic low mood [35,36]. These mechanisms are particularly reflected in depression, which affects overall mood regulation over time, whereas anxiety responses to competition and uncertainty may be common across all generations—potentially explaining the lack of a generational difference in anxiety symptoms.

The correlation between long working hours and depressive symptoms was approximately three-fold stronger in the MZ generation than in the non-MZ generation, demonstrating a significant generational difference in vulnerability. Several factors may underlie this result. First, MZ workers strongly value work–life balance and personal happiness. For them, long work hours represent not only a simple physical burden but also an infringement on core values, thereby intensifying emotional burnout and depression, as described in the Job Demands–Resources Model [37,38]. Second, the MZ generation has relatively less job control, economic stability, and social resources because they are in the earlier stages of their careers, whereas non-MZ workers are more likely to benefit from higher rank and greater autonomy [39]. Third, baseline CES-D scores were already higher in the MZ group, consistent with previous studies showing increased depression among younger cohorts facing challenges such as fierce competition, job insecurity, economic pressures, and comparison culture amplified by SNS [40,41]. In this context, the additional burden of long work hours likely accelerates depressive symptoms in MZ workers. Finally, from the perspective of Person–Environment Fit theory, generational differences in job values and organizational culture may explain this pattern. MZ participants are more likely to perceive long work hours as a poor fit with organizational expectations, thereby reinforcing depressive outcomes.

Although there were no significant generational differences in mean CUXOS anxiety scores in our study, the effects of long working hours were much greater in the MZ generation. This pattern differed from depression, suggesting that MZ workers do not necessarily experience more anxiety than previous generations at baseline, but that longer working hours tend to rapidly exacerbate their anxiety symptoms. Several explanations are possible. First, the MZ generation tends to perceive uncertainty about the future and a loss of control more strongly when the boundaries between work and personal life are blurred [42,43], which can heighten anxiety under conditions of extended work hours. Second, as the first generation continually exposed to real-time social comparisons through SNS, MZ workers may experience intensified psychological pressure, and their anxiety responses may become overactivated when these stressors are combined with long work hours. In contrast, non-MZ workers are more likely to have secured job stability, organizational influence, and social resources in the later stages of their careers, potentially buffering the impact of longer work hours. Older workers may instead perceive role responsibility or physical fatigue as major stressors rather than anxiety [44], which could also lead to underreporting of anxiety on self-report questionnaires. In summary, these findings highlight that generational differences shape vulnerabilities to anxiety and that customized approaches are required for managing both depression and anxiety in workplace mental health.

Although the three-way interactions with sex and marital status did not reach conventional significance, their consistent direction suggested that the detrimental impact of long working hours might be particularly pronounced among younger female and unmarried workers. These exploratory patterns align with previous findings that MZ women tend to experience greater emotional exhaustion, work–life imbalance, and relational strain. Taken together, these results highlight the need for generation- and gender-sensitive approaches in workplace mental health policies.

Our study had several limitations. First, “generation” is closely correlated with age, years of work, and income, making it difficult to completely distinguish whether the observed effects reflect purely generational differences or differences associated with life stage and work experience. Although we controlled for major demographic variables, a significant interaction remained between generation and work hours, suggesting that unique generational values or experiences contributed to the effect. Second, the explanatory power of the models was modest (R^2^ = 7–8%), indicating that many non–work-related and individual-level factors—such as personality traits, resilience, and informal support—also influence mental health but were not included in this dataset. However, since the primary objective of this study was to examine the moderating effect of generation, the strong significance of the interaction term supports the intended analysis (*p* < 0.001) provides sufficient evidence for the hypothesized effects. Third, the cross-sectional design precluded causal inference. Although previous longitudinal research has consistently demonstrated the harmful consequences of extended working hours, our findings highlight generation as an additional and previously underexplored moderating factor. Fourth, reliance on self-report measures may have introduced reporting bias, which could differ across generations. Moreover, the use of self-report measures such as the CES-D and CUXOS may have limited our ability to detect subtle generational differences in symptom expression. Younger employees may report psychological distress more openly or interpret items differently due to changing attitudes toward mental health and workplace culture, whereas older generations might underreport symptoms owing to stigma or differing health perceptions. Thus, some intergenerational variation may not have been fully captured by these instruments. Future research should employ longitudinal designs, incorporate objective indices, and include qualitative methods to further explore mechanisms of generational vulnerability. Finally, the cultural and organizational context of South Korea may limit the generalisability of our findings. Korean workplaces are often characterized by long working hours, hierarchical relationships, and strong collectivist norms emphasizing group harmony and endurance. These cultural and structural factors may amplify stress responses to extended work hours compared with more individualistic or flexible work cultures. Therefore, caution should be exercised when applying these results to other sociocultural or occupational contexts.

## 5. Conclusions

Using multicenter data from 11,372 Korean employees, this study demonstrated that long working hours exacerbate both depression and anxiety, with effects differing significantly by generation. Standardized scales (CES-D and CUXOS) and rigorous statistical analyses (mean centering, interaction testing, stratified analysis) showed that the negative effects of extended working hours were approximately three-fold greater in the MZ generation than in the non-MZ generation. This suggests that longer working hours may increase emotional burden in MZ workers because they conflict with the core generational value of work–life balance. These findings call for fundamental reflection on current human resource management strategies and organizational culture, which often neglect individual and generational characteristics. Sustainable corporate growth depends on employee well-being. Accordingly, new leadership approaches and workplace systems are needed that acknowledge and respect generational differences. This study provides empirical evidence to support the development of healthier, more productive workplaces founded on mutual understanding across generations and offers an important basis for workplace mental health policies and cultural reforms.

## Figures and Tables

**Table 1:** Baseline characteristics in participants.

Characteristics	Overall (*n* = 11,372)	Non-MZ (*n* = 3834)	MZ (*n* = 7538)	*p*-Value
Age (years), mean ± SD	36.72 ± 9.43	47.70 ± 5.08	31.14 ± 5.34	<0.001
Sex				<0.001
Male, *n* (%)	7737 (68.0)	2775 (72.4)	4962 (65.8)	
Female, *n* (%)	3635 (32.0)	1059 (27.6)	2576 (34.2)	
Education (graduate)				<0.001
College graduate or below, *n* (%)	2759 (24.3)	907 (23.7)	1852 (24.6)	
University graduate, *n* (%)	6837 (60.1)	1950 (50.9)	4887 (64.8)	
Master’s degree or higher, *n* (%)	1776 (15.6)	977 (25.5)	799 (10.6)	
Marital status				<0.001
Married, *n* (%)	6366 (56.0)	3389 (88.4)	2977 (39.5)	
Unmarried, *n* (%)	4808 (42.3)	313 (8.2)	4498 (59.6)	
Other, *n* (%)	198 (1.7)	132 (3.4)	66 (0.9)	
Years of service (years), mean ± SD	10.55 ± 9.09	19.10 ± 9.57	6.20 ± 4.68	<0.001
Hours of work per week (hours), mean ± SD	46.82 ± 7.53	46.14 ± 8.34	47.17 ± 7.06	<0.001
Monthly earned income (million won)				<0.001
Less than 3 million won, *n* (%)	2949 (25.9)	354 (9.2)	2595 (34.4)	
3–4 million won, *n* (%)	3046 (26.8)	639 (16.7)	2407 (31.9)	
Over 4 million won, *n* (%)	4660 (41.0)	2571 (67.1)	2089 (27.7)	
Missing, *n* (%)	717 (6.3)	270 (7.0)	447 (5.9)	
Clinical characteristics				
CES-D score, mean ± SD	17.08 ± 14.59	13.50 ± 9.63	14.99 ± 10.16	<0.001
CUXOS score, mean ± SD	16.85 ± 5.42	16.85 ± 14.14	17.19 ± 14.82	0.230

SD, standard deviation; CES-D, Center for Epidemiologic Studies Depression Scale; CUXOS, Clinically Useful Anxiety Outcome Scale; MZ generation, participants born on or after 1 January 1980.

**Table 2:** Multivariable linear regression for depressive symptoms (CES-D).

Predictor	Model 1				Model 2			
	B	SE	t	*p*-Value	B	SE	t	*p*-Value
Age	0.018	0.017	1.020	0.308	0.098	0.022	4.511	<0.001
Sex (Female)	3.834	0.205	18.744	<0.001	3.984	0.203	19.639	<0.001
Education (University graduate)	1.255	0.234	5.362	<0.001	1.121	0.232	4.827	<0.001
Education (Master’s degree or higher)	−0.087	0.334	−0.260	0.795	−0.032	0.330	−0.097	0.923
Marital status (Unmarried)	1.629	0.244	6.687	<0.001	1.514	0.242	6.252	<0.001
Marital status (Other)	3.827	0.707	5.411	<0.001	3.941	0.700	5.629	<0.001
Years of service	0.018	0.016	1.124	0.261	0.032	0.016	1.981	0.048
Income (3–4 million won)	0.232	0.262	0.886	0.375	−0.131	0.260	−0.504	0.614
Income (Over 4 million won)	−0.560	0.278	−2.011	0.044	−0.943	0.277	−3.407	<0.001
Working hour (centered)					0.080	0.019	4.249	<0.001
MZ generation					1.942	0.352	5.524	<0.001
Working hour (centered) × MZ generation					0.140	0.025	5.710	<0.001
R^2^	0.051				0.071			
F	61.028 **				66.400 **			

CES-D, Center for Epidemiologic Studies Depression Scale; B, estimate of the regression coefficient; SE, standard error; R^2^, explanatory power; MZ generation, participants born on or after 1 January 1980. Sex–Male, Education–College graduate or below, Marital status—Married, Income—Less than 3 million won were set as the reference groups for the model; ** *p* < 0.001.

**Table 3:** Multivariable linear regression for anxiety symptoms (CUXOS).

Predictor	Model 1				Model 2			
	B	SE	t	*p*-Value	B	SE	t	*p*-Value
Age	0.098	0.025	3.930	<0.001	0.204	0.032	6.465	<0.001
Sex (Female)	6.783	0.296	22.887	<0.001	6.992	0.294	23.764	<0.001
Education (University graduate)	2.273	0.339	6.703	<0.001	2.094	0.337	6.215	<0.001
Education (Master’s degree or higher)	−0.028	0.483	−0.058	0.954	0.047	0.479	0.098	0.922
Marital status (Unmarried)	−0.036	0.353	−0.103	0.918	−0.197	0.351	−0.561	0.575
Marital status (Other)	2.616	1.025	2.553	0.011	2.779	1.016	2.736	0.006
Years of service	0.014	0.023	0.597	0.551	0.032	0.024	1.354	0.176
Income (3–4 million won)	0.209	0.379	0.550	0.582	−0.284	0.378	−0.751	0.452
Income (Over 4 million won)	−1.692	0.403	−4.195	<0.001	−2.214	0.401	−5.516	<0.001
Working hour (centered)					0.119	0.027	4.363	<0.001
MZ generation					2.526	0.510	4.954	<0.001
Working hour (centered) × MZ generation					0.182	0.036	5.126	<0.001
R^2^	0.063				0.080			
F	75.766 **				75.867 **			

CUXOS, Clinically Useful Anxiety Outcome Scale; B, estimate of the regression coefficient; SE, standard error; R^2^, explanatory power; MZ generation, participants born on or after 1 January 1980. Sex–Male, Education–College graduate or below, Marital status—Married, Income—Less than 3 million won were set as the reference groups for the model; ** *p* < 0.001.

## Data Availability

The data necessary to interpret, replicate, and build upon the findings of this study are available from the corresponding author, S.J.C., upon reasonable request. The data are not publicly available because of ethical restrictions related to patient privacy and consent.

## Supplementary Materials

The following supporting information can be downloaded at: https://www.mdpi.com/article/10.3390/healthcare13233002/s1.

## Abbreviations

CES-D	Center for Epidemiologic Studies Depression Scale
CUXOS	Clinically Useful Anxiety Outcome Scale
MZ	Millennial and Generation Z (participants born on or after 1 January 1980)
KRW	Korean Won
SPSS	Statistical Package for the Social Sciences
SD	Standard Deviation
R^2^	Coefficient of Determination (R-squared)
VIF	Variance Inflation Factor
