# Supplementary material for: The Moderating Effect of Generation on the Association Between Long Working Hours and Mental Health: A Cross-Sectional Study of Korean Employees
Source: Healthcare (Basel). 2025 Nov 21;13(23):3002. doi: 10.3390/healthcare13233002 (PMC12692718; doi:10.3390/healthcare13233002)
Supplement: Supplementary file 1 [file healthcare-13-03002-s001.zip › healthcare-3882624-supplementary-Table S1.S2.pdf]

Table S1. Multivariable linear regression for depressive symptoms (CES-D) among non MZ and MZ generation.

Predictor	Non MZ				MZ			
	B	SE	t	p-value	B	SE	t	p-value
Age	-0.170	0.034	-5.025	<0.001	0.223	0.029	7.607	<0.001
Gender (Female)	2.432	0.357	6.811	<0.001	4.295	0.254	16.917	<0.001
Education (University graduate)	0.173	0.383	0.451	0.652	1.342	0.301	4.458	<0.001
Education (Master's degree or higher)	-0.142	0.464	-0.306	0.759	-0.033	0.473	-0.070	0.944
Marital status (Unmarried)	3.564	0.572	6.229	<0.001	2.072	0.286	7.237	<0.001
Marital status (Other)	3.794	0.836	4.536	<0.001	4.870	1.214	4.013	<0.001
Years of service	0.064	0.019	3.363	<0.001	0.070	0.032	2.224	0.026
Income (3-4 million won)	-0.219	0.631	-0.347	0.729	-0.357	0.291	-1.224	0.221
Income (Over 4 million won)	-2.159	0.566	-3.818	<0.001	-1.088	0.339	-3.213	0.001
Working hour (centered)	0.064	0.018	3.485	<0.001	0.223	0.016	13.882	<0.001
R^2	0.061				0.085			
F	22.486**				63.930**			

CES-D, Center for Epidemiologic Studies Depression Scale; B, estimate of the regression coefficient; SE, standard error; R^2, explanatory power; MZ generation, participants born on or after January 1, 1980.
Gender – Male, Education – College graduate or below, Marital status - Married, Income – Less than 3 million won were set as the reference groups for the model.

Table S2. Multivariable linear regression for anxiety symptoms (CUXOS) among non MZ and MZ generation.

Predictor	Non MZ				MZ			
	B	SE	t	p-value	B	SE	t	p-value
Age	-0.127	0.049	-2.573	0.010	0.365	0.042	8.594	<0.001
Gender (Female)	5.055	0.524	9.652	<0.001	7.425	0.367	20.219	<0.001
Education (University graduate)	0.899	0.562	1.600	0.110	2.292	0.435	5.266	<0.001
Education (Master's degree or higher)	-0.255	0.680	-0.374	0.708	0.089	0.684	0.131	0.896
Marital status (Unmarried)	3.287	0.839	3.917	<0.001	0.107	0.414	0.259	0.796
Marital status (Other)	3.089	1.227	2.517	0.012	3.245	1.755	1.849	0.065
Years of service	0.092	0.028	3.306	<0.001	0.018	0.046	0.388	0.698
Income (3-4 million won)	-1.232	0.926	-1.330	0.183	-0.375	0.421	-0.889	0.374
Income (Over 4 million won)	-3.966	0.830	-4.781	<0.001	-2.434	0.490	-4.969	<0.001
Working hour (centered)	0.100	0.027	3.725	<0.001	0.305	0.023	13.158	<0.001
R^2	0.063				0.100			
F	23.461**				75.817**			

CUXOS, Clinically Useful Anxiety Outcome Scale; B, estimate of the regression coefficient; SE, standard error; R^2, explanatory power; MZ generation, participants born on or after January 1, 1980.
Gender – Male, Education – College graduate or below, Marital status - Married, Income – Less than 3 million won were set as the reference groups for the model.
