# Supplementary figures and images for: The Moderating Effect of Generation on the Association Between Long Working Hours and Mental Health: A Cross-Sectional Study of Korean Employees
Source: Healthcare (Basel). 2025 Nov 21;13(23):3002. doi: 10.3390/healthcare13233002 (PMC12692718; doi:10.3390/healthcare13233002)

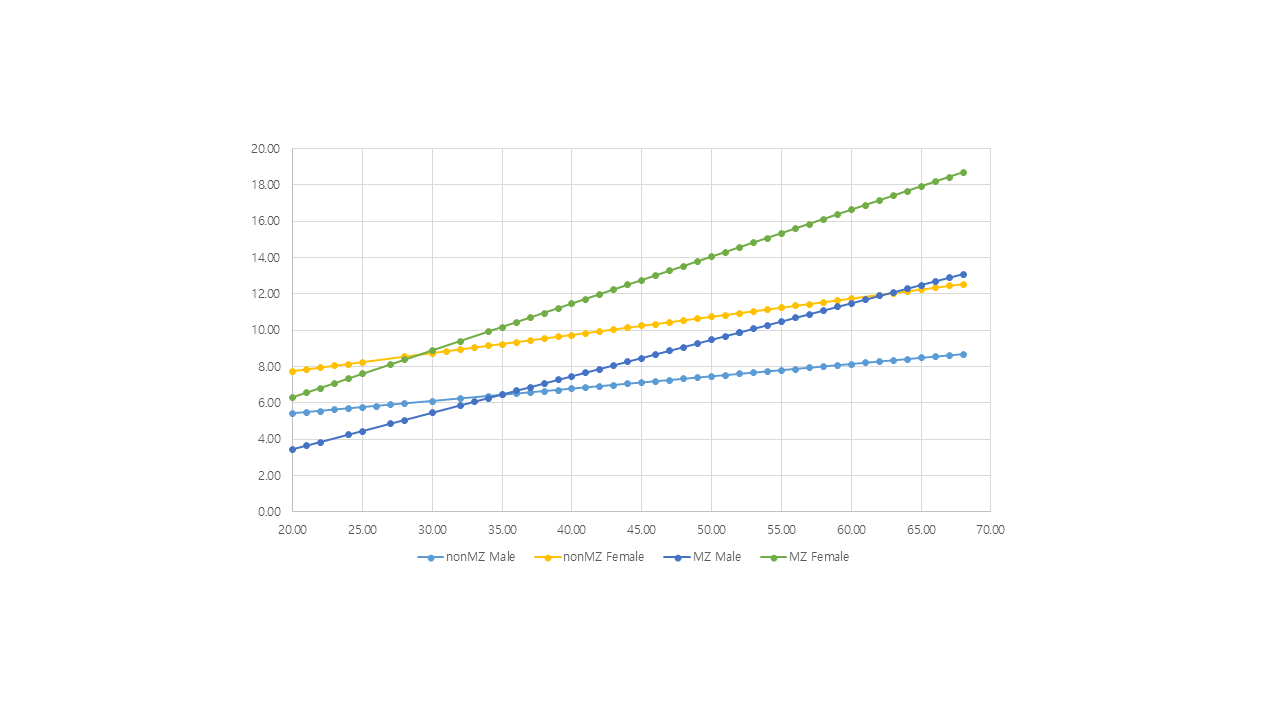

Supplement: Supplementary file 1 [file healthcare-13-03002-s001.zip › Supplementray Figures/Supplementary Figure S1.TIF]

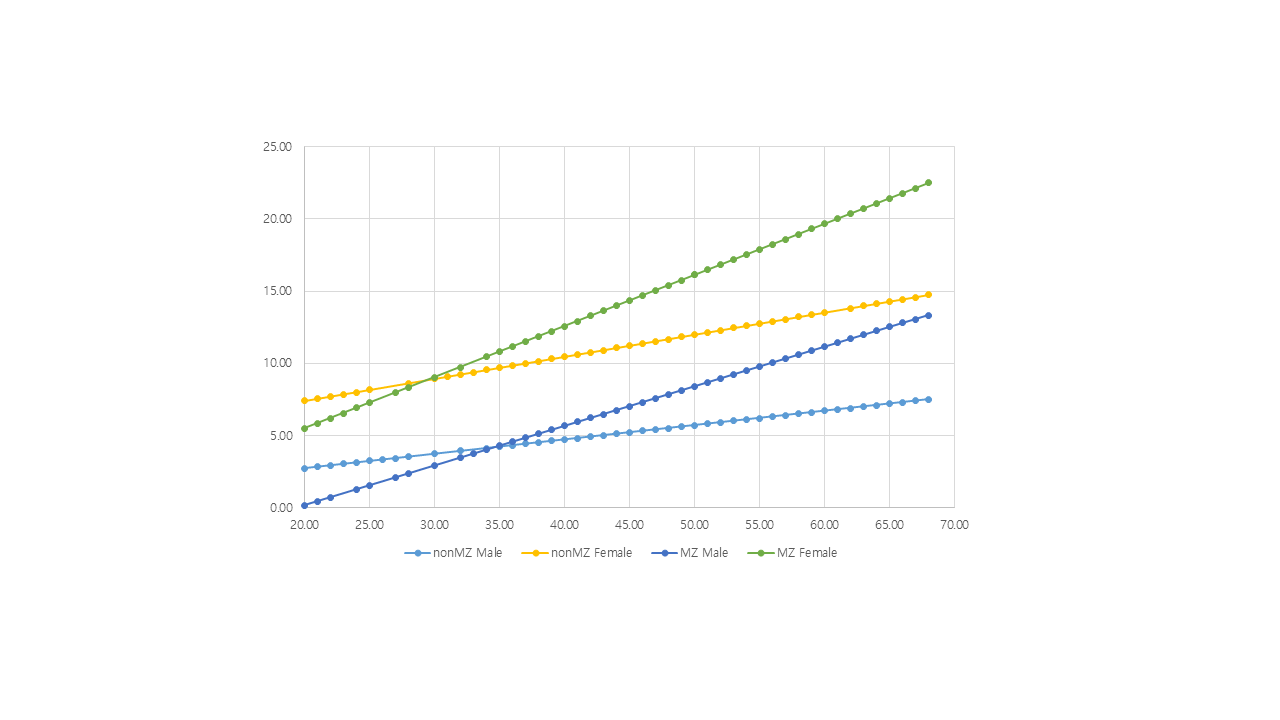

Supplement: Supplementary file 1 [file healthcare-13-03002-s001.zip › Supplementray Figures/Supplementary Figure S2.TIF]

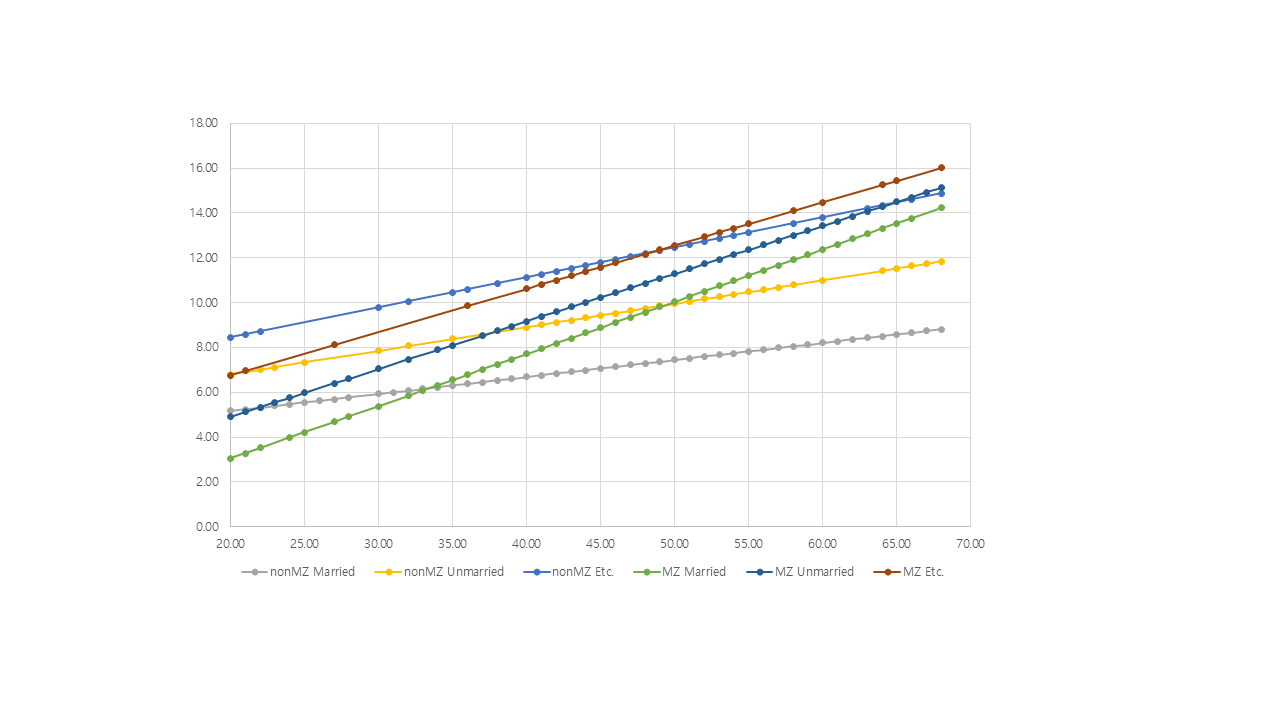

Supplement: Supplementary file 1 [file healthcare-13-03002-s001.zip › Supplementray Figures/Supplementary Figure S3.TIF]

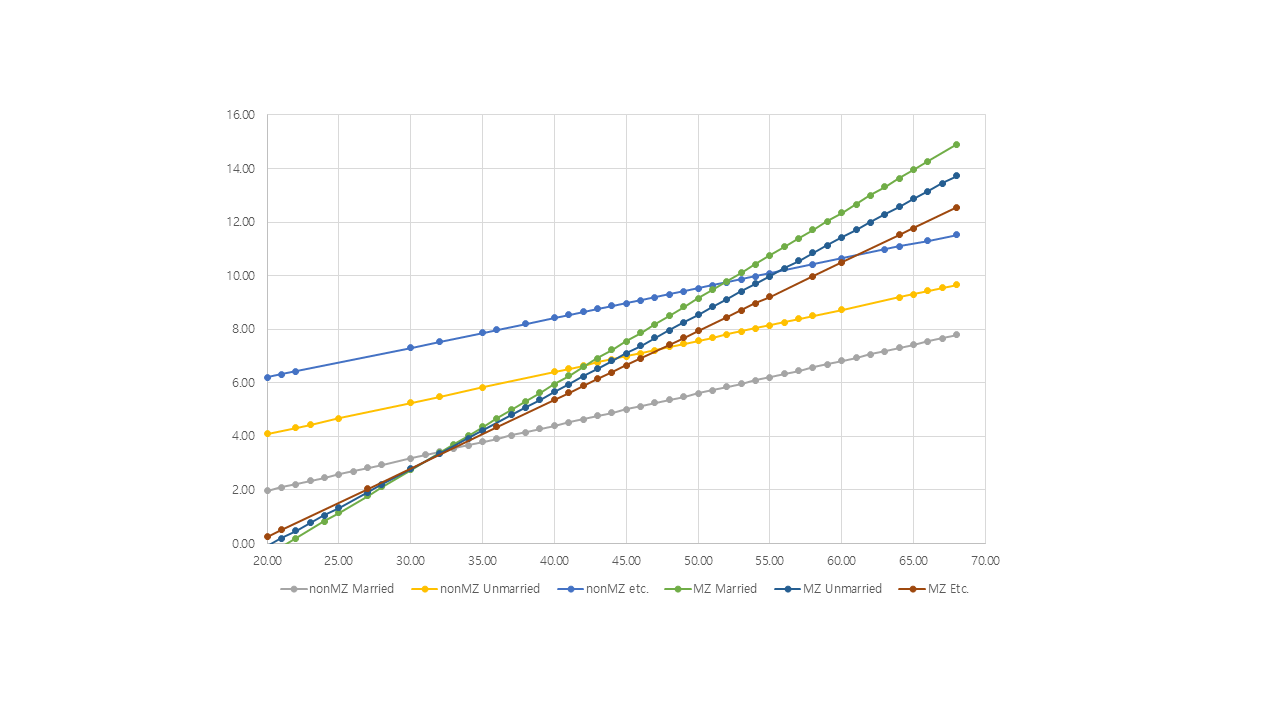

Supplement: Supplementary file 1 [file healthcare-13-03002-s001.zip › Supplementray Figures/Supplementary Figure S4.TIF]

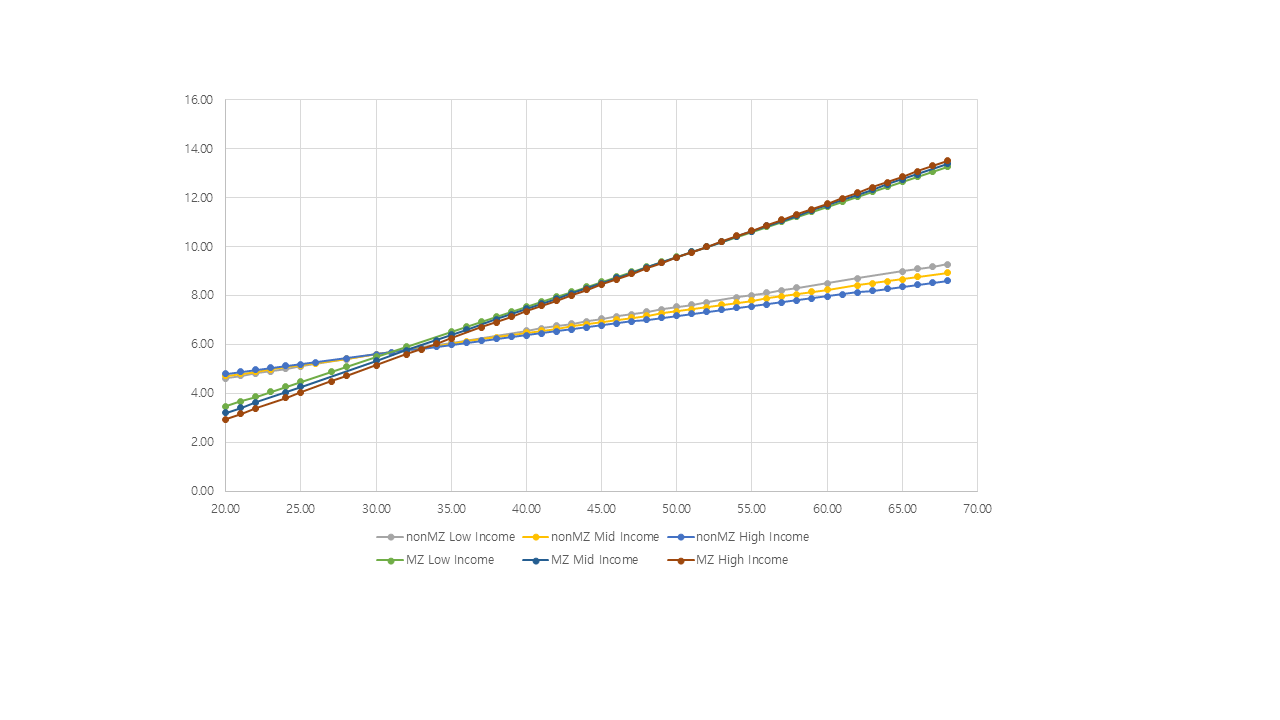

Supplement: Supplementary file 1 [file healthcare-13-03002-s001.zip › Supplementray Figures/Supplementary Figure S5.TIF]

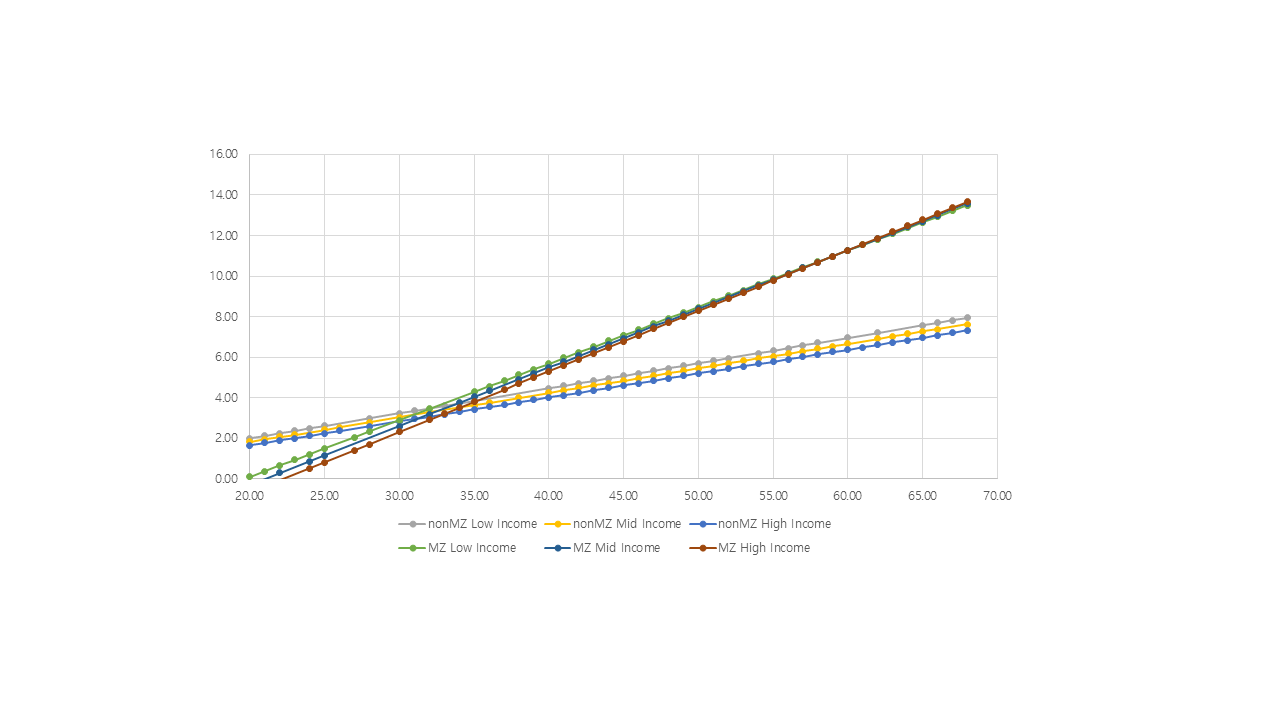

Supplement: Supplementary file 1 [file healthcare-13-03002-s001.zip › Supplementray Figures/Supplementary Figure S6.TIF]
